# Inflammatory response gene polymorphisms and their relationship with colorectal cancer risk

**DOI:** 10.1186/1471-2407-8-112

**Published:** 2008-04-23

**Authors:** Janina Suchy, Ewa Kłujszo-Grabowska, Józef Kładny, Cezary Cybulski, Dominika Wokołorczyk, Jolanta Szymańska-Pasternak, Grzegorz Kurzawski, Rodney J Scott, Jan Lubiński

**Affiliations:** 1 International Hereditary Cancer Center-Department of Genetics and Pathology, Pomeranian Medical University, Szczecin, Poland; 2 Department of General and Oncological Surgery, Pomeranian Medical University, Szczecin, Poland; 3 Discipline of Medical Genetics, Faculty of Health, University of Newcastle and the Hunter Medical Research Institute, Newcastle, NSW, Australia

## Abstract

**Backgroud:**

Patients with chronic inflammatory bowel disease (IBD) are at an increased risk of colorectal cancer (CRC) and it is estimated that one in six persons diagnosed with IBD will develop CRC. This fact suggests that genetic variations in inflammatory response genes may act as CRC disease risk modifiers.

**Methods:**

In order to test this hypothesis we investigated a series of polymorphisms in 6 genes (NOD2, DLG5, OCTN1, OCTN2, IL4, TNFα) associated with the inflammatory response on a group of 607 consecutive newly diagnosed colorectal cancer patients and compared the results to controls (350 consecutive newborns and 607 age, sex and geographically matched controls).

**Results:**

Of the six genes only one polymorphism in TNFα(-1031T/T) showed any tendency to be associated with disease risk (64.9% for controls and 71.4% for CRC) which we further characterized on a larger cohort of CRC patients and found a more profound relationship between the TNFα -1031T/T genotype and disease (64.5% for controls vs 74.7% for CRC cases above 70 yrs). Then, we investigated this result and identified a suggestive tendency, linking the TNFα -1031T/T genotype and a previously identified change in the CARD15/NOD2 gene (OR = 1.87; p = 0,02 for CRC cases above 60 yrs).

**Conclusion:**

The association of polymorphisms in genes involved in the inflammatory response and CRC onset suggest that there are genetic changes capable of influencing disease risk in older persons.

## Background

Biological and epidemiological data indicate a clear association between chronic inflammation and malignancy [1]. Patients with inflammatory bowel disease (IBD), including Crohn disease (CD) and ulcerative colitis (UC), are at increased risk of developing colorectal cancer [2]. Epidemiological and linkage studies strongly suggest the involvement of genetic factors in IBD, especially those associated with inflammation.

Recently, it has been shown that the NOD2/CARD15 (nucleotide oligomerisation domain 2/caspase activating recruitment domain 15) gene is implicated in the susceptibility to Crohn's disease [3,4]. NOD2/CARD15 is expressed intracellularly in monocytes/macrophages and granulocytes, where it activates the nuclear factor NF-κB, making it responsive to bacterial lipopolysaccharides which culminate in the activation of apoptosis [5]. Patients harbouring mutant NOD2 alleles are presumably deficient in the activation of NF-κB and consequently the appropriate response to bacterial infection [5-7]. Among patients with CD, the frequency of C-insertion mutation in exon 11 of NOD2 is about three times higher compared to control populations [4].

In a previous investigation, we found a significant association between the NOD2 frameshift constitutional mutation (3020insC) and the risk of colorectal cancer in patients diagnosed above the age of 50 years (OR = 2.23; p = 0.0046) [8].

The association between IBD and polymorphisms in other inflammation-related genes involved in the innate immune response like DLG5 [9], IL-4 [10], OCTN [11], TNFα [12] have been reported but their relationship to disease risk requires further investigation. Together, the associations between polymorphisms in inflammatory response genes and IBD make them attractive candidate susceptibility genes for colorectal cancer since approximately 1:6 individuals with IBD will develop malignancy [13]. In addition, there is sufficient evidence to suggest that remodeling of the immune system with age may also alter cancer risk [14] and consequently alterations in the activity of TNFα may become more pronounced with age.

In the present study, we evaluated whether single nucleotide polymorphisms (SNPs) within a number of inflammatory response genes are associated with an increased risk of colorectal cancer. Ten SNPs in the following six genes: NOD2 (2140 C/T, 2722 G/C), DLG5 (113 G/A), OCTN1 (1672 C/T), OCTN2 (-207 G/C), IL4 (-590 C/T) and TNFα (-308 G/A, -857 C/T, -863 C/A, -1031 T/C) were screened in a consecutive series of colorectal cancer patients using a two-staged approach.

## Methods

The investigation was performed in two stages, first to pre-select candidate genetic variations by determinating the frequency of any alteration in an unselected colorectal cancer population compared to the newborn controls. The second, depth analysis was performed on specifically matched control subjects to the colorectal cancer group.

For the first stage of the study a group of 350 consecutive, newly diagnosed collected patients affected by colorectal adenocarcinomas from the clinical hospital SPSK-2 Szczecin and a group of 350 control subjects (consecutive newborns from the clinical hospitals of Szczecin) were studied.

During the second stage of the investigation a further 257 consecutive, newly diagnosed colorectal cancer (CRC) patients from Szczecin were combined with the first 350 CRC patients to give a final 607 cases and compared to 607 age, year of birth, sex and geographically matched healthy controls from families negative for a cancer family history. The CRC patients – 316 males and 291 females – were collected between the years 1996 and 2006 with the average age of disease diagnosis being 63.2 (range 27–92 yrs). Definite diagnosis of Crohn disease has not been established in any CRC patients.

The adult controls were initially identified during the population genetic cancer screening programme performed in West-Pomerania from 2000–2001. Family doctors and community nurses collected questionnaires with cancer family histories from 1 258000 (85%) of the inhabitants. Before inclusion in the study, all selected adults were verified for their pedigree/clinical data at cancer genetic outpatient clinics that were part of our centre.

This investigation was approved by the Institutional Ethics review committee of the Pomeranian Academy of Medicine, Szczecin and informed consent was obtained from all participants prior to enrolment into the study.

DNA samples were extracted from peripheral blood lymphocytes derived from CRC patients and matched controls or from umbilical cord blood of newborns according to method of Miller et al [15].

Cases and controls were genotyped by using the PCR-RFLP method described by others for the following SNPs: TNFα -857, -863, -1031 [16], TNFα -308 [17], IL-4 -590C/T [18], NOD2 2140C/T, 2722G/C [19], 3020insC [8], DLG5113G/A [9]. Genotyping of OCTN1 1672C/T was performed using the following primers F: TAGTCTGACTGTCCTGATTGGAAGC, R: TGCTTATTCTCCCTAAGGCATTTTGG followed by AluI restriction fragment length polymorphism (RFLP) analysis.

For OCTN2 -207G/C polymorphism analysis the primers F: CCGCTCTGCCTGCCAGCGGG, R: CAAGACCGTCCGCGGAGGGTAGG and a HpaII RFLP were used. For each SNP studied a random number were taken and subjected to direct sequencing analysis to reduce the risk that the genotypes were a result of genotyping error. The concordance between the samples taken for DNA sequencing compared to PCR-RFLP analysis was 100 percent. If samples failed to amplify after three attempts the sample and its partner were left out of the analysis such that all analyses were performed on matched pairs.

Statistical analysis included a comparison of the prevalence of the genotype frequency in cases versus controls. Odds ratios were generated from two-by-two contingency tables and statistical significance was determined using Fisher's exact test.

Each genetic variant studied was in Hardy-Weinberg equilibrium.

To estimate the genotype/disease interaction the patient group was stratified by age and logistic regression modelling was undertaken [31]. All interaction analyses were based on the genotype comparison using a codominant model.

## Results

The study was performed in two stages to ensure that the Polish population frequencies of the chosen polymorphisms were no different to that reported in the NCBI SNP database [32].

During the first stage of the study ten polymorphisms in six genes were analysed and no significant differences in the frequencies of nine of the variants was observed in the CRC group compared to the newborn control population. Only one polymorphism (TNFα -1031 T/T) showed a tendency towards being over-represented in the CRC group compared to the newborn control population. The frequency of the TNFα -1031 T/T genotype was 71.4% in the CRC group against 64.9% in the newborn control population.

To better define the relationship between the TNFα -1031T/T SNP and cancer risk a further 257 consecutively collected CRC patients were assayed for the presence of the T/T genotype and the results compared to an adult control population that has been matched for year of birth, age, sex and domicile (Table 1). The results reveal that the -1031T/T genotype in the CRC group overall when compared to the control population was not significantly different in its frequency (71.0% vs 67.7%).

**Table 1:** Screening of polymorphisms tested for association with CRC between cases and sex, age and geographically matched controls.

**Gene/Change**	**Age groups**	**Genotype**	**Matched controls n = 607**	**CRC cases n = 607**	**OR (95% CI)**	**p-value**
NOD2/3020insC	consecutive	WT/WT	558 (91.9%)	544 (89.6%)	1.00	0.065
		WT/insC	49 (8.1%)	60 (9.9%)	1.26 (0.85–1.87)	
		insC/insC	0 (0%)	3 (0.5%)	NA	
	≤ 50	WT/WT	91 (91.9%)	94 (95%)	1.00	0.38
		WT/insC	8 (8.1%)	5 (5%)	0.60 (0.19–1.92)	
	>50	WT/WT	467 (91.9%)	450 (88.6%)	1.00	**0.038**
		WT/insC	41 (8.1%)	55 (10.8%)	**1.39 **(0.91–2.14)	
		insC/insC	0 (0%)	3 (0.6%)	NA	
	>60	WT/WT	350 (91.4%)	335 (87.5%)	1.00	**0.042**
		WT/insC	33 (8.6%)	45 (11.8%)	**1.43 **(0.89–2.29)	
		insC/insC	0 (0%)	3 (0.8%)	NA	
	>70	WT/WT	168 (90.3%)	159 (85.5%)	1.00	0.15
		WT/insC	18 (9.7%)	27 (14.5%)	**1.59 **(0.84–3.01)	
						
TNFα/-1031 T/C	consecutive	T/T	411 (67.7%)	431 (71%)	1.00	0.14
		T/C	170 (28%)	161 (26.5%)	0.89 (0.69–1.15)	
		C/C	26 (4.3%)	15 (2.5%)	0.54 (0.28–1.04)	
	≤50	T/T	71 (71.7%)	63 (63.6%)	1.00	0.42
		T/C	24 (24.2%)	32 (32.3%)	1.53 (0.81–2.90)	
		C/C	4 (4%)	4 (4%)	1.19 (0.28–5.01)	
	>50	T/T	340 (66.9%)	368 (72.4%)	1.00	**0.04**
		T/C	146 (28.7%)	129 (25.4%)	0.80 (0.61–1.06)	
		C/C	22 (4.3%)	11 (2.2%)	**0.45 **(0.22–0.95)	
	>60	T/T	252 (65.8%)	276 (72.1%)	1.00	0.059
		T/C	115 (30%)	99 (25.9%)	0.76 (0.55–1.05)	
		C/C	16 (4.2%)	8 (2.1%)	**0.44 **(0.18–1.05)	
	>70	T/T	120 (64.5%)	139 (74.7%)	1.00	0.084
		T/C	60 (32.3%)	43 (23.1%)	0.61 (0.38–0.96)	
		C/C	6 (3.2%)	4 (2.1%)	0.57 (0.15–2.08)	

When the consecutively collected CRC group was stratified for age of diagnosis, there appeared to be an association of the TNFα -1031T/T SNP with later ages of disease diagnosis (see Table 1).

Previously we have shown in the Polish population that the NOD2 3020insC mutation is associated with CRC risk [8], which remained in this new analysis (Table 1). A combined analysis of patients harbouring the NOD2 mutation and the TNFα -1031T/T SNP was performed. The combined analysis was performed on 607 CRC patients and 607 matched healthy controls in order to determine if the TNFα -1031 T/T SNP acts in association with the NOD2 3020insC change. From this analysis a total of 41 cases were identified in the CRC group compared to 27 in the control group, which revealed an over-representation of the two SNPs in the CRC group compared to the control group (OR 1.50, p = 0.45). The average age of disease onset in persons harbouring both polymorphisms compared to those only harbouring the TNFα -1031T/T change was found to 69.8 years of age (range 48–84) compared to non-carriers 65.3 years of age (range 30–92), which was not statistically significant. Conversely, we observed a trend toward a protective effect in that there was an under-representation of the TNFα -1031 C/C and NOD2 3020insC negative carriers in the CRC population (OR 0.49, p = 0.45) (see Table 2).

**Table 2:** Combined analysis of TNFα -1031T/C and NOD2 3020insC in consecutively collected CRC cases compared to matched control subjects.

	**NOD2 WT/WT**			**NOD2 WT/insC + insC/insC**		
	
**Gene**	**Controls**	**CRC**	**OR (95%CI)**	**Controls**	**CRC**	**OR (95% CI)**
TNFα T/T	384	390	1.00	27	41	**1.50 **(0.90–2.49)
TNFα T/C	150	142	0.93 (0.71–1.22)	20	19	0.94 (0.49–1.79)
TNFα C/C	24	12	**0.49 **(0.24–1.00)	2	3	1.48 (0.25–8.97)

p = 0.45

When stratified by age, the association between the TNFα -1031 T/T and NOD2 3020insC carriers and disease became more apparent (see Table 3) with age thereby providing further evidence that these two polymorphisms are associated with later onset disease.

**Table 3:** Frequency of the combined genotypes (TNFα-1031T/C & NOD2 3020insC) and changes in the OR with increasing age of diagnosis in consecutively collected colorectal cancer patients.

		**NOD2 WT/WT**			**NOD2 WT/insC + insC/insC**		
		
**Age groups**	**Gene**	**Controls**	**CRC**	**OR (95% CI)**	**Controls**	**CRC**	**OR (95% CI)**
≤ 50	**TNFα T/T**	65	61	1.00	6	2	**0.34 **(0.07–1.80)
	**TNFα T/C**	23	29	1.36 (0.71–2.62)	1	3	3.18 (0.32–31.47)
	**TNFα C/C**	3	4	1.44 (0.31–6.70)	1	0	0.00

p = 0.22							

>50	**TNFα T/T**	319	329	1.00	21	39	**1.80 **(1.04–3.14)
	**TNFα T/C**	127	113	0.86 (0.64–1.16)	19	12.6	0.82 (0.41–1.62)
	**TNFα C/C**	21	8	0.37 (0.16–0.84)	1	3	2.95 (0.30–28.58)

p = 0.12							

>60	**TNFα T/T**	236	245	1.00	16	31	**1.87 **(1.00–3.52)
	**TNFα T/C**	98	85	0.83 (0.59–1.17)	17	14	0.80 (0.38–1.65)
	**TNFα C/C**	16	5	0.29 (0.11–0.82)	0	3	---

**p = 0.02**							

>70	**TNFα T/T**	111	119	1.00	9	20	**2.08 **(0.91–4.76)
	**TNFα T/C**	51	37	0.67 (0.41–1.11)	9	6	0.63 (0.22–1.83)
	**TNFα C/C**	6	3	0.46 (0.11–1.89)	0	1	---

p = 0.24							

## Discussion

In the present study, we compared the frequency of a series of polymorphisms in different inflammatory response genes associated with IBD (DLG5, IL-4, OCTN, TNFα and NOD2) in colorectal cancer cases against controls. The results indicate that the TNFα -1031 TT variant frequency had a tendency to be over-represented in the CRC population compared to an unmatched newborn control population and compared to stringently matched controls. In addition, the association observed in the CRC group compared to the matched controls became more apparent when the CRC patients were stratified by age. The increasing association of the TNFα -1031 T/T genotype with the age of diagnosis of colorectal cancer suggests that there is a link between age-related remodeling of the immune system [14] which results in a greater predisposition to disease with aging in the presence of this polymorphism.

Other polymorphisms were also investigated within the TNFα promoter region of the gene (-857 C/T, -308 G/A, -238 G/A) and they appeared to confer no protective influence on colorectal cancer risk, a finding which is consistent with other observations [20-23]. Nevertheless associations of the TNFα promoter -1031 polymorphism together with the -308 and -238 polymorphisms have also been made with respect to invasive breast carcinoma [24] and an increased risk of gastric cancer [25]. Other malignancies have also been investigated but no significant differences in the distribution of the -1031 T/T variant have been found in nasopharyngeal carcinoma [26] or in malignant melanoma [27]. There is no information about the relationship between the TNFα -1031 T/T polymorphism and colorectal cancer and further studies are necessary to confirm above results.

We have previously reported an association between the NOD2 3020insC mutation and colorectal cancer risk [8] which has been confirmed in two other reports [28,29] but remains controversial as two other reports failed to identify any association [13,30]. In this current report, instead of using general population controls to determine whether the frequency of the NOD2 3020insC mutation was over-represented in our CRC population we used an age, year of birth, sex and domicile matched control population and identified a similar association between this mutation and disease. An age dependent trend was also observed for CRC patients over 50 years of age.

By undertaking a combined analysis of carriers of the NOD2 3020insC mutation who have the TNFα -1031T/T genotype and comparing their frequency in the CRC population to the matched control population a more pronounced interaction emerges. There appears to be an over-representation of NOD2 3020insC and TNFα -1031T/T carriers in the CRC group compared to the matched control group suggesting that it is the combined effect of the two polymorphisms that is particularly important and that their influence becomes more profound with age as evidenced by the increasing over-representation of the two polymorphisms in older CRC patients.

Our studies have several obvious limitations: the size of the study population is relatively small and some stratifications were performed that increased the likelihood of false positive outcomes as a result of the multiple comparison testing. Finally, additional larger investigations using other larger patient groups in ours as well as other populations are required to unequivocally determine the role of these SNPs on CRC risk.

## Conclusion

In conclusion, it appears that independently both the TNFα-1031T/T and the NOD2 3020insC polymorphisms may act as low risk modifiers of colorectal cancer risk. Together, the evidence presented in this report suggests an additive effect of the two polymorphisms that becomes increasingly important with advancing age with respect to the risk of CRC. Studies on a larger cohort of colorectal cancer cases and matched controls are necessary to clarify the relationship between the TNFα -1031T/T and NOD2 3020insC polymorphisms and CRC.

## Competing interests

The authors declare that they have no competing interests.

## Authors' contributions

JS participated in the design of the study, carried out the molecular genetic studies, performed the statistical analysis and drafted the manuscript. EK-G participated in the design of the study, carried out the molecular genetic studies and performed the statistical analysis, both authors contributed equally to this work. JK carried out the clinical genetic studies. CC, DW, JS-P carried out the molecular genetic studies. GK conceived of the study, participated in its design. RJS contributed to interpretation of data, coordination and helped to draft the manuscript. JL conceived of the study, participated in its design and coordination and helped to draft the manuscript. All authors read and approved the final manuscript.

## Pre-publication history

The pre-publication history for this paper can be accessed here:



## Supplementary Material

Additional file 1 Genotype distribution of ten SNPs analysed in a group of 350 CRC cases and 350 controls (newborns). The data provided report the numbers for each genotype of the SNPs analysed in the control and cases group. Click here for file
