# Supplementary material for: Inflammatory response gene polymorphisms and their relationship with colorectal cancer risk
Source: BMC Cancer. 2008 Apr 23;8:112. doi: 10.1186/1471-2407-8-112 (PMC2386482; doi:10.1186/1471-2407-8-112)
Supplement: Additional file 1 — Genotype distribution of ten SNPs analysed in a group of 350 CRC cases and 350 controls (newborns). The data provided report the numbers for each genotype of the SNPs analysed in the control and cases group. [file 1471-2407-8-112-S1.doc]

Genotype distribution of ten SNPs analysed in a group of 350 CRC cases and 350 controls (newborns)

| **Gene/Change** | **CRC**  **n=350** | **%** | **Controls**  **n=350** | **%** | **OR (%CI)** | **p** |
| --- | --- | --- | --- | --- | --- | --- |
| NOD2/2140 C/T |  |  |  |  |  |  |
| CC | 325 | 92.9 | 325 | 92.9 | 1 |  |
| CT | 25 | 7.1 | 25 | 7.1 | 1 (0.56-1.77) | 1.11 |
| TT | 0 | 0 | 0 | 0 |  |  |
| NOD2/2722 G/C |  |  |  |  |  |  |
| GG | 341 | 97.4 | 343 | 98.0 | 1 |  |
| GC | 9 | 2.6 | 7 | 2.0 | 1.29 (0.47-3.51) | 0.80 |
| CC | 0 | 0 | 0 | 0 |  |  |
| DLG5/113 G/A |  |  |  |  |  |  |
| GG | 281 | 80.3 | 287 | 82.0 | 1 |  |
| GA | 69 | 19.7 | 59 | 16.9 | 1.19 (0.81-1.75) | 0.37 |
| AA | 0 | 0 | 4 | 11.4 | 0.11 (0.006-2.11) | 0.12 |
| OCTN1/1672 C/T |  |  |  |  |  |  |
| CC | 132 | 37.7 | 148 | 42.3 | 1 |  |
| CT | 180 | 51.4 | 159 | 45.4 | 1.27 (0.92-1.74) | 0.14 |
| TT | 38 | 10.9 | 43 | 12.3 | 0.99 (0.60-1.62) | 1.00 |
| OCTN2/-207 G/C |  |  |  |  |  |  |
| GG | 115 | 32.9 | 116 | 33.1 | 1 |  |
| GC | 164 | 46.9 | 168 | 48.0 | 0.98 (0.70-1.37) | 0.93 |
| CC | 71 | 20.2 | 66 | 18.9 | 1.08 (0.71-1.65) | 0.74 |
| IL4/-509 C/T |  |  |  |  |  |  |
| CC | 225 | 64.3 | 230 | 65.7 | 1 |  |
| CT | 113 | 32.3 | 107 | 30.6 | 1.08 (0.78-1.49) | 0.68 |
| TT | 12 | 3.4 | 13 | 3.7 | 0.94 (4.42-2.11) | 1.00 |
| TNFα/-308 G/A |  |  |  |  |  |  |
| GG | 254 | 72.6 | 248 | 70.9 | 1 |  |
| GA | 87 | 24.9 | 95 | 27.1 | 0.89 (0.63-1.25) | 0.54 |
| AA | 9 | 2.5 | 7 | 2.0 | 1.25 (0.46-3.42) | 0.80 |
| TNFα/-857 C/T |  |  |  |  |  |  |
| CC | 253 | 72.3 | 242 | 69.1 | 1 |  |
| CT | 88 | 25.1 | 98 | 28.0 | 0.85 (0.61-1.20) | 0.39 |
| TT | 9 | 2.6 | 10 | 2.9 | 0.86 (0.34-2.15) | 0.81 |
| TNFα/-863 C/A |  |  |  |  |  |  |
| CC | 262 | 74.9 | 257 | 73.4 | 1 |  |
| CA | 77 | 22.0 | 83 | 23.7 | 0.91 (0.63-1.29) | 0.65 |
| AA | 11 | 3.1 | 10 | 2.9 | 1.07 (0.45-2.58) | 1.00 |
| TNFα/-1031 T/C |  |  |  |  |  |  |
| TT | 250 | 71.4 | 227 | 64.9 | 1 |  |
| TC | 90 | 25.7 | 107 | 30.6 | 0.76 (0.54-1.06) | 0.12 |
| CC | 10 | 2.9 | 16 | 4.5 | 0.56 (0.25-1.27) | 0.22 |
